# Pregnancy and Fibromyalgia and their Interrelationships: A Systematic Review

**DOI:** 10.31138/mjr.220424.pam

**Published:** 2025-03-31

**Authors:** Jozélio Freire de Carvalho, Thelma L. Skare

**Affiliations:** 1 Núcleo de Pesquisa em Doenças Crônicas não Transmissíveis (NUPEN), School of Nutrition from the Federal University of Bahia, Salvador, Bahia, Brazil;; 2 Rheumatology Unit, Hospital Evangélico Mackenzie, Curitiba, PR, Brazil

**Keywords:** fibromyalgia, chronic pain, pregnancy

## Abstract

**Background:**

Fibromyalgia (FM) occurs frequently in women of childbearing age. These patients may become pregnant, and it is essential to know the influence of pregnancy on this disease.

**Aim:**

To review the studies of pregnancy in FM.

**Methods:**

To systematically search for articles on pregnancy and FM between 1966 and April 2024. No language limitation was used. Scielo, PubMed, and Embase databases were analysed.

**Results:**

Twelve articles with 8,833 patients were found. Patients’ ages varied from > 18 to 65 years old. FM patients had a lower number of children and more nulliparity than controls. Studies that analysed FM symptoms during pregnancy found symptom worsening, mainly pain, anxiety, depression, and gestational diabetes, were found to be more common than in controls in 2 papers. Regarding neonatal outcomes, only four studies evaluated these data: two of them found that FM had no adverse effect on the neonate’s health. In contrast, the other two found that these babies were more likely to be premature, to have intrauterine growth restriction, and to have low Apgar.

**Conclusion:**

This systematic review demonstrates that pregnancy in FM usually has a bad prognosis since obstetric and FM outcomes are worse during this period. Results of repercussions on the offspring are controversial.

## INTRODUCTION

Fibromyalgia (FM) is a condition characterised by diffuse chronic pain associated with anxiety, depression, irritable bowel syndrome, paraesthesia, perfectionism, and low quality of life. It is a prevalent disease, affecting about 5% of the population worldwide.^1^ Although its pathophysiologic mechanisms are not fully understood, it is known that there is an interplay among central nervous system neural activity, small fibers neuropathy, abnormal metabolism of biogenic amines, and immunologic factors.^2^ FM is commonly treated with physical exercise, psychotherapy, and antidepressant and/or anticonvulsant drugs such as duloxetine, pregabalin, and others.^1^

Most patients with FM are females of childbearing age, raising questions about the influence of pregnancy on this disease. Sexual hormones seem to be associated with alterations in pain threshold and pain tolerance,^3^ and this may interfere with FM symptoms during this period. Moreover, pregnancy itself is commonly as- sociated with joint and muscle pain. Progesterone is considered neuroprotective and antinociceptive,^4^ while prolactin has a pronociceptive effect.^5^ Oestrogen fluctuations have been shown to increase pain intensity and perception.^6^ Moreover, serotonin - a neurotransmitter associated with feelings of happiness, vitality, and euphoria and postulated to be decreased in FM,^7^ is lower during gestation, decreasing as pregnancy progresses.^8^ Nevertheless, its relationship to FM symptoms in pregnancy has not been well studied. In addition. depression and anxiety, commonly found in FM patients, have been associated with lower birth weight and small gestational age babies.^9,10^ The objective of this article is to perform a systematic review of the studies that evaluated the relationship between pregnancy and FM.

## MATERIALS AND METHODS

### Literature review

We performed a systematic search of articles published in PubMed/MEDLINE, EMBASE, and Scielo from 1966 to April 2024 using the following MeSH entry terms: “fibromyalgia” AND “pregnancy.” We used equivalent strategies in other databases. No language restriction was established. The reference lists in the selected articles were analysed to identify other publications.

Initially, two authors (JFC and TLS) performed the literature search and independently selected the study abstracts. In the second stage, the same reviewers independently read the full-text articles selected by abstracts. The authors followed PRISMA guidelines.^11^

A standardised form to extract the following information from relevant articles was designed: authors, year of publication, number of patients studied, study design, demographic data, pregnancy influencing FM evolution, FM influencing pregnancy development, and baby outcomes.

Exclusion criteria were *in vivo* and *in vitro* studies, Editorials and review articles.

We used the Jadad score to evaluate the quality of the studies.

## RESULTS

**Figure 1.**
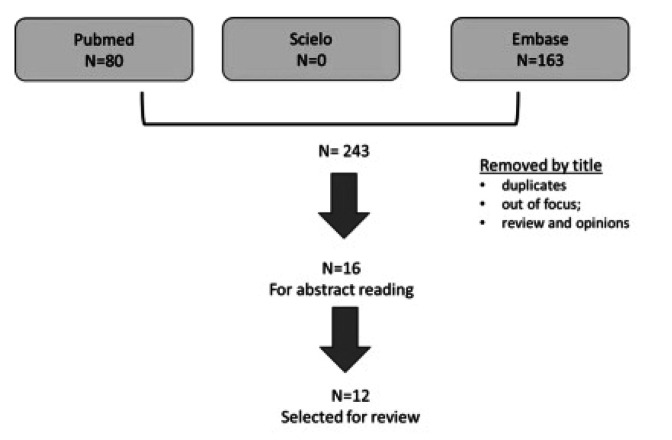
Selection of articles for revision on fibromyalgia and pregnancy.

The flow chart with the search results is in **Figure 1**.

Twelve articles were published in this field, with 8,833 patients, were identified.^12–23^ The study designs were cross-sectional (n=6) [12–17], retrospective (n=3),^18–20^ and prospective (n=3).^21–23^ In eight of them, controls were used.^13–16,19,22,23^ The countries in which the studies were performed are: Turkey (n=3), Germany (n=2), Israel (n=1), the United States (n=1), Egypt (n=1), Canada (n=1); Ivory Coast (n=1), Iraq (n=1) and Norway (n=1). The age of the studied patients varied from > 18 to 65 years old.^13^

Two authors observed worsening of FM symptoms during pregnancy.^18,20^ Gestational diabetes was more frequent in FM patients in two studies^19,20^; pregnancy anxiety, anxiety about childbirth,^22^ depression during pregnancy,^20,23^ and postpartum^18,22^ were also more frequent in the FM group.

Females with FM had higher rates of caesarean section in two studies^17,20^ but not in a third one^19^; Morsy et al.^15^ observed that these patients had more severe headaches after lumbar puncture for caesarean section anaesthesia.

Two papers^19,20^ observed intrauterine growth restriction. Babies’ Apgar scores were lower in one study^21^ but similar to controls in another.^19^ Ostensen et al.^18^ described no adverse effects on neonates’ health.

More FM patients have never been pregnant^13,14^ and have had fewer pregnancies than non-FM patients,^12^ although in one study, the number of pregnancies was similar.^23^ According to Raphael et al.,^12^ FM patients may choose to have fewer children or engage in behaviours that lead to fewer children.

Regarding quality of the studies, 8/12 (67%) had a good Jadad score (≥ 3) and 4/12 (33%) had a non-good quality Jadad score.

Details of selected works can be seen in **Table 1**.

**Table 1.** Studies on pregnancy and fibromyalgia.

**Author, year**	**Study design**	**Country**	**N**	**Age**	**Fibromyalgia criteria**	**Obstetric outcomes**	**FM patient outcomes**	**Neonatal outcomes**	**Jadad score**
Ostensen et al., 1997^18^	Retrospective	Norway	26 women with 40 pregnancies	Median: 38 yo	1990 ACR criteria	-	All but one experienced worsening of FM during pregnancy, mainly in the last trimester. After 6 months of delivery, 4 females felt better, and 33 worsened. Increase in depression and anxiety in the postpartum period. Hormonal changes associated with abortion, contraceptive use, and breastfeeding did not modulate symptom severity. Pre-menstrual worsening of symptoms in 72% of FM.	No adverse effect on the health of the neonate.	3
Raphael et al., 2000^13^	Cross-sectional, case-control	USA	162 white females with MFP; 173 controls.	Range: 18–65 yo		-	Controls and patients with MFP had an exact number of pregnancies, several females who had never been pregnant rate, and abortions. The subgroup of patients with FM had a lower number of pregnancies.	-	3
Schochat et al., 2003^14^	Cross-sectional, case-control.	Germany	36 FM; 44 with WPI without FM; 408 controls.	Range: 35–74 yo	1990 ACR criteria	-	FM, but not patients only with WPI, had significantly later menarche (OR=2.2 for >14 years) and had never been pregnant (OR=0.3).	-	2
Klingmann et al., 2008^21^	Prospective	Germany	93 FM	51.4 yo	ND	-	FM patients with a shorter gestational length (<38 weeks) showed a lower cortisol awakening response than those with >38 weeks. > 70% reported severe psychological stress alone or in combination with other factors at disease onset.	-	2
Zioni et al., 2011^19^	Retrospective, case-control.	Israel	Deliveries: - 112 FM - 487 controls	> 19 yo	ND	FM had ↑ rates of IUGR, recurrent abortions, gestational DM, and polyhydramnios. Lower rates of preterm deliveries. No differences in rates of cesarean deliveries.	-	No differences in low Apgar scores at 1 and 5 min.	2
Atasever et al., 2016^22^	Prospective, case-control	Turkey	277 pregnant females: −150 FM −127 controls	26.5 yo	2010 ACR criteria	-	FM had more pregnancy anxiety, anxiety about childbirth, postpartum anxiety, more depression, and worse physical function. FM had lower serotonin levels than controls; serotonin serum levels decreased as pregnancy progressed. Negative correlations between serotonin levels and WPI, VAS pain, PFS, and total FIQ questions.	-	3
Tulay et al., 2016^23^	Prospective	Turkey	-111 FM -76 controls	Mean=39.0 yo	2010 ACR criteria	-	Depression was higher in the FM group. Both groups were similar regarding the number of pregnancies, parity, and duration of breastfeeding.	-	3
Morsy et al., 2016^15^	Cross sectional- case-control	Egypt	140 pregnant females with elective caesarean section - 70 FM - 70 non-FM	24 yo	1990 ACR criteria	-	FM had more post-dural puncture headaches.	-	3
Genç et al., 2016^71^	Cross-sectional case-control	Turkey	360 pregnantfemales: -136 FM; -224 controls.	Mean=26.5 yo	2010 ACR criteria	-	FM had higher pain, fatigue, muscle weakness and muscle pain, headache, chest pain, abdominal cramps, dizziness, constipation, nausea, frequent urination, waking unfreshened, cognitive disturbances, depression, anxiety, and fear of childbirth compared to controls.	-	3
Magtanong et al., 2017^20^	Population-based retrospective cohort study	Canada	7,758 (0.06%) FM out of 12,584.918 births	84% were ≥25 yo	Inernational Classification of Diseases −9 edition.	Women with FM were at greater risk of gestational diabetes, preterm premature rupture of membranes, placental abruption and intrauterine growth restriction, caesarean deliveries, births complicated by venous thromboembolism, more extended hospital stay, and less labour induction.	FM was more commonly older, overweight or obese, hypertensive, and users of alcohol, tobacco, and illicit drugs. They have more anxiety, depression, and bipolar disorder.	Infants are more likely to be premature and have intrauterine growth restriction.	3
Shakir et al., 2018^13^	Cross-sectional	Iraq	202 infertile females	ND	1990 ACR criteria and 2012 Canadian Guidelines	FM was present in 23.8% of the infertile sample; FM had a longer duration of infertility; FM did not associate with the type of infertility (primary or secondary)			2
Koné et al., 2022^17^	Cross-sectional	Ivory Coast	271 pregnant females who came for child delivery (131 with FM)	ND	ND		FM had more caesarean sections	FM newborns were more unhealthy babies (Apgar ≤ 8)	3

ACR: American College of Rheumatology; FM: fibromyalgia; NA: not available; ND: not described; yo: years old; WPI: Widespread Pain Index; VAS: visual analogic scale; FIQ: fibromyalgia impact questionnaire; PFS: physical function scale; IUGR: intrauterine growth restriction; DM: diabetes mellitus.

## DISCUSSION

Fibromyalgia is a disease with profound repercussions on patients’ quality of life. The constant experience of pain, sleep disturbances, limitation of motor performance, and lack of fitness limits the ability to perform ordinary daily activities.^18^ Although pregnancy and childbirth are among the most critical events in a woman’s life, they may enhance physical strain, aggravating FM symptoms. Pregnancy is also a susceptible period for females’ psychosocial condition, with a risk of deteriorating depression and/or negative mood symptoms previously found.^18^ Atasever et al.^22^ observed that FM patients had more pregnancy concerns and more anxiety about delivery than controls. Genç et al. also noted the fear of childbirth.^16^

Furthermore, even in normal females, pregnancy may be associated with fatigue and sleep disturbances,^24^ which are well-known symptoms of FM; musculoskeletal pain may appear or be aggravated due to the loosening of ligaments and joints throughout the body.^25,26^ Also, hormonal changes may alter the perception of pain. It has been postulated that oestrogens have significant properties regulating pain by acting on intracellular receptors, modifying gene expression, and G-coupled proteins found in the peripheral and central nervous systems.^27^ Moreover, neural pathways related to the modulation of pain, such as noradrenergic, serotonergic, dopaminergic, and endogenous opioid pathways, are affected by oestrogens. In general, nociception is considered to be increased by oestrogens.^6^ Placental neurohormones may also play a role in this context. The human placenta is an endocrine organ that produces and releases several signalling substances, including cytokines, neuropeptides, neurosteroids, and amines,^28^ that may modulate pain perception. Curiously, studies in animal models with neuritis and treatment with culture-expanded placenta-derived adherent cells have shown that they may have therapeutic effects on neuropathic pain.^29^

Fear of harming the baby has led to treatment interruption in almost half of the patients in work by Østensen et al.,^18^ and this may further aggravate the symptoms. In the studies by Raphael et al.,^12^ Schochat et al.,14 and Shakin et al.,^13^ it was observed that the number of nulliparous females was higher in females with FM and that FM patients had fewer children. Although none of the authors were able to determine the risk factors for this finding, one explanation is that volitional factors may be involved in reduced fecundity as a consequence of living with pain.

To the authors’ knowledge, this is the first study to systematically review the relationships between pregnancy and FM. Some limitations are observed: for instance, no metanalysis was possible due to the heterogeneity of the variables studied per article. Also, no reviewed articles referred to treating fibromyalgia during pregnancy, and research on this area is urgently needed to help FM patients during this particular period of their lives. Moreover, the prognosis for future pregnancies based on the present data unfortunately is unknown. It was not studies yet. It would be an interesting point to be studies in future trials. Another suggestion for future research is the subgroup analysis in studies with larger number of participants.

## CONCLUSION

This systematic review suggests that patients with FM who become pregnant are at increased risk of symptoms worsening, especially pain, anxiety, depression, and gestational diabetes. In addition, an increased risk of adverse outcomes in pregnancy is also observed, in two studies the frequencies of prematurity, intrauterine growth restriction, and low Apgar were higher in FM women. Therefore, clinicians should alert and instruct their FM patients regarding pregnancy and recommend a careful obstetric follow-up.

## CONFLICT OF INTEREST

The authors declare that they have no conflict of interest.

## References

[1] Clauw DJ. Fibromyalgia: an overview. Am J Med 2009; 122(12 Suppl): S3–SS13. doi: 10.1016/j.amjmed.2009.09.006. 19962494

[2] Di Carlo M Bianchi B Salaffi F Pellegrino G Iannuccelli C Giorgi V Fibromyalgia: one year in review 2024. Clin Exp Rheumatol 2024 Apr 10. doi: 10.55563/clinexprheumatol/mbyi1n. 38607678

[3] Okifuji A Turk DC. Sex hormones and pain in regularly menstruating women with fibromyalgia syndrome. J Pain 2006;7(11):851–859. doi: 10.1016/j.jpain.2006.04.005. 17074627

[4] Szewczyk AK Ulutas S Aktürk T Al-Hassany L Börner C Cernigliaro F European Headache Federation School of Advanced Studies (EHF-SAS). Prolactin and oxytocin: potential targets for migraine treatment. J Headache Pain 2023;24(1):31. doi: 10.1186/s10194-023-01557-6. 36967387 PMC10041814

[5] Hornung RS Raut NG Cantu DJ Lockhart LM Averitt DL. Sigma-1 receptors and progesterone metabolizing enzymes in nociceptive sensory neurons of the female rat trigeminal ganglia: A neural substrate for the antinociceptive actions of progesterone. Mol Pain 2022; 18:17448069211069255. doi: 10.1177/17448069211069255. 35040378 PMC8777333

[6] Athnaiel O Cantillo S Paredes S Knezevic NN. The role of sex hormones in pain-related conditions. Int J Mol Sci 2023; 24(3):1866. doi: 10.3390/ijms24031866. 36768188 PMC9915903

[7] Berger M Gray JA Roth BL. The expanded biology of serotonin. Annu Rev Med 2009;60:355–66. doi: 10.1146/annurev.med.60.042307.110802. 19630576 PMC5864293

[8] Fuchs D Schrocksnadel H Baier-Bitterlich G Dapunt O Wachter H. Activated cellular immunity and decreased serum tryptophan in a healthy pregnancy. Adv Exp Med Biol 1996;398:149–53. doi: 10.1007/978-1-4613-0381-7_24. 8906258

[9] Marcus SM. Depression during pregnancy: rates, risks, and consequences—Motherisk Update 2008. Can J Clin Pharmacol 2009;16: e15–e22.PMID: 19164843. 19164843

[10] Maina G Saracco P Giolito MR Danelon D Bogetto F Todros T. Impact of maternal psychological distress on fetal weight, prematurity and intrauterine growth retardation. J Affect Disord 2008;111:214–220. doi: 10.1016/j.jad.2008.02.017. 18394713

[11] Page MJ McKenzie JE Bossuyt PM Boutron I Hoffmann TC Mulrow CD The PRISMA 2020 statement: an updated guideline for reporting systematic reviews. BMJ 2021;372:n71. doi: 10.1136/bmj.n71. 33782057 PMC8005924

[12] Raphael KG Marbach JJ. Comorbid fibromyalgia accounts for reduced fecundity in women with myofascial face pain Clin J Pain 2000;16(1):29–36. doi: 10.1097/00002508-200003000-00006. 10741816

[13] Shakir RA Badr AH Jassim NA Hummadi JA. Prevalence of fibromyalgia among a sample of infertile women: A cross-sectional study from Baghdad, Iraq. J Fac Med Baghdad 2018;60(4):228–33. doi: 10.32007/jfacmedbagdad.604700.

[14] Schochat T Beckmann C. [Sociodemographic characteristics, risk factors and reproductive history in subjects with fibromyalgia--results of a population-based case-control study]. Z Rheumatol 2003;62(1):46–59. doi: 10.1007/s00393-003-0447-5. 12624804

[15] Morsy KM Osman AM Shaaban OM El-Hammady DH. Post dural puncture headache in fibromyalgia after cesarean section: A comparative cohort study. Pain Physician 2016;19(6):E871–6.PMID: 27454277. 27454277

[16] Genç H Atasever M Duyur Çakit B Seval M Koç A. the effects of fibromyalgia syndrome on physical function and psychological status of pregnant females. Arch Rheumatol 2017;32(2):129–40. doi: 10.5606/ArchRheumatol.2017.6028. 30375568 PMC6190985

[17] Koné CM Kambiré NA Kouakou K Yapi A. Fibromyalgia of women who gave birth and pregnancy outcome parameters. Open J Epidemiol 2022;12(01):1–11. doi: 10.4236/ojepi.2022.121001.

[18] Ostensen M Rugelsjøen A Wigers SH. The effect of reproductive events and alterations of sex hormone levels on the symptoms of fibromyalgia. Scand J Rheumatol. 1997;26(5):355–60. doi: 10.3109/03009749709065698. 9385346

[19] Zioni T Buskila D Aricha-Tamir B Wiznitzer A Sheiner E. Pregnancy outcome in patients with fibromyalgia syndrome. J Matern Fetal Neonatal Med 2011; 24(11):1325–8. doi: 10.3109/14767058.2010.551152. 21284491

[20] Magtanong GG Spence AR Czuzoj-Shulman N Abenhaim HA. Maternal and neonatal outcomes among pregnant women with fibromyalgia: a population-based study of 12 million births. J Matern Fetal Neonatal Med 2019; 32(3):404–10. doi: 10.1080/14767058.2017.1381684. 28954564

[21] Klingmann PO Kugler I Steffke TS Bellingrath S Kudielka BM Hellhammer DH. Sex-specific prenatal programming: a risk for fibromyalgia? Ann N Y Acad Sci 2008;1148:446–55. doi: 10.1196/annals.1410.020. 19120140

[22] Atasever M Namlı Kalem M Sönmez Ç Seval MM Yüce T Sahin Aker S. Lower serotonin level and higher rate of fibromyalgia syndrome with advancing pregnancy. J Matern Fetal Neonatal Med 2017; 30(18):2204–11. doi: 10.1080/14767058.2016.1243096. 27696917

[23] Tulay KT Emrullah T Aydin A Ciledag OF. The effect of fibromyalgia syndrome to gravidity, parity and duration of breastfeeding; A prospective study from Turkey. Pak J Med Sci 2016;32(3):545–9. doi: 10.12669/pjms.323.9574. 27375686 PMC4928395

[24] Tsai SY Lin JW Kuo LT Thomas KA. Daily sleep and fatigue characteristics in nulliparous women during the third trimester of pregnancy. Sleep 2012;35(2):257–62. doi: 10.5665/sleep.1634. 22294816 PMC3250365

[25] Casagrande D Gugala Z Clark SM Lindsey RW. Low back pain and pelvic girdle pain in pregnancy. J Am Acad Orthop Surg 2015;23:539–49. doi: 10.5435/JAAOS-D-14-00248. 26271756

[26] Choi HJ Lee JC Lee YJ Lee EB Shim SS Park JS Prevalence and clinical features of arthralgia/arthritis in healthy pregnant women. Rheumatol Int 2008;28(11):1111–5. doi: 10.1007/s00296-008-0596-6. 18548252

[27] Manolagas SC Kousteni S. Perspective: Nonreproductive Sites of action of reproductive hormones. Endocrinology 2001;142:2200–4. doi: 10.1210/endo.142.6.8221. 11356663

[28] De Bonis M Torricelli M Severi FM Luisi S De Leo V Petraglia F. Neuroendocrine aspects of placenta and pregnancy. Gynecol Endocrinol 2012;28:22–6. doi: 10.3109/09513590.2012.651933. 22394300

[29] He S Khan J Gleason J Eliav E Fik-Rymarkiewicz E Herzberg U Placenta-derived adherent cells attenuate hyperalgesia and neuroinflammatory response associated with perineural inflammation in rats. Brain Behav Immun 2013;27(1):185–92. doi: 10.1016/j.bbi.2012.10.015. 23103445

